# A 3-mRNA-based prognostic signature of survival in oral squamous cell carcinoma

**DOI:** 10.7717/peerj.7360

**Published:** 2019-07-31

**Authors:** Ruoyan Cao, Qiqi Wu, Qiulan Li, Mianfeng Yao, Hongbo Zhou

**Affiliations:** 1 Department of Prosthodontics, Xiangya Stomatological Hospital & School of Stomatology, Central South University, Changsha, China; 2 Department of Endodontics, Xiangya Stomatological Hospital & School of Stomatology, Central South University, Changsha, China; 3 Department of Stomatology, The Second Xiangya Hospital, Central South University, Changsha, China; 4 Department of Stomatology, Xiangya Hospital, Central South University, Changsha, China

**Keywords:** Bioinformatics, mRNA, Prognosis, Oral squamous cell carcinoma

## Abstract

**Background:**

Oral squamous cell carcinoma (OSCC) is the most common type of head and neck squamous cell carcinoma with an unsatisfactory prognosis. The aim of this study was to identify potential prognostic mRNA biomarkers of OSCC based on analysis of The Cancer Genome Atlas (TCGA).

**Methods:**

Expression profiles and clinical data of OSCC patients were collected from TCGA database. Univariate Cox analysis and the least absolute shrinkage and selection operator Cox (LASSO Cox) regression were used to primarily screen prognostic biomarkers. Then multivariate Cox analysis was performed to build a prognostic model based on the selected prognostic mRNAs. Nomograms were generated to predict the individual’s overall survival at 3 and 5 years. The model performance was assessed by the time-dependent receiver operating characteristic (ROC) curve and calibration plot in both training cohort and validation cohort (GSE41613 from NCBI GEO databases). In addition, machine learning was used to assess the importance of risk factors of OSCC. Finally, in order to explore the potential mechanisms of OSCC, Kyoto Encyclopedia of Genes and Genomes (KEGG) pathway analysis was completed.

**Results:**

Three mRNAs (*CLEC3B*, *C6* and *CLCN1*) were finally identified as a prognostic biomarker pattern. The risk score was imputed as: (−0.38602 × expression level of *CLEC3B*) + (−0.20632 × expression level of *CLCN1*) + (0.31541 × expression level of *C6*). In the TCGA training cohort, the area under the curve (AUC) was 0.705 and 0.711 for 3- and 5-year survival, respectively. In the validation cohort, AUC was 0.718 and 0.717 for 3- and 5-year survival. A satisfactory agreement between predictive values and observation values was demonstrated by the calibration curve in the probabilities of 3- and 5- year survival in both cohorts. Furthermore, machine learning identified the 3-mRNA signature as the most important risk factor to survival of OSCC. Neuroactive ligand-receptor interaction was most enriched mostly in KEGG pathway analysis.

**Conclusion:**

A 3-mRNA signature (*CLEC3B*, *C6* and *CLCN1*) successfully predicted the survival of OSCC patients in both training and test cohort. In addition, this signature was an independent and the most important risk factor of OSCC.

## Introduction

Oral squamous cell carcinoma (OSCC) is a common malignant tumor, which results in approximately over 600,000 new cases and 350,000 related deaths every year (Siegel, Miller & Jemal, 2017). Advances in treatments for OSCC have significantly improved the quality of life and further life expectancy of patients. However, the overall clinical outcomes of patients remain poor, especially among those diagnosed at advanced stages (Shimomura et al., 2018; Wang et al., 2017). Hence, exploring effective prognostic models is essential to predict overall survival (OS) of OSCC patients and guide the therapeutic process for clinicians. Present prognostic models of OSCC focus on multiple clinicopathological parameters like age, smoking status, and advanced clinical stage at diagnosis (Boxberg et al., 2018; Lee et al., 2010). However, cancers generally possess complex molecular regulation mechanisms and thus traditional predictive factors are limited by their efficiency, specificity, and consistency among patients (Shen et al., 2017a).

Recently, molecular biomarkers of diagnosis or prognosis, such as protein-coding genes and non-coding RNAs, have gained much attentions in oncology. Messenger RNAs (mRNAs) have been highlighted for their important roles in physiological and pathological processes as well as their potential predictive abilities (Feng et al., 2019; Tschirdewahn et al., 2019). Moreover, an integrated model composed of multiple genes has been shown to be more predictive than single clinic biomarker (Shao et al., 2018). An area under the curve (AUC) of 0.7 and above indicates relatively good prediction. For example, a 7-mRNA signature (*AATF*, *APP*, *GNPDA1*, *HPRT1*, *LASP1*, *P4HA1* and *ILF3*) of head and neck squamous cell carcinoma (HNSCC) shows a moderate predictive ability for 5-year OS (AUC for training set, 0.75; testing set, 0.66) (Shen et al., 2017a). Another prognostic model of HNSCC based on 6-mRNA (*FOXL2NB*, *PCOLCE2*, *SPINK6*, *ULBP2*, *KCNJ18* and *RFPL1*) also has a good performance of 5-year OS (AUC for training set, 0.766; testing set, 0.669) (Tian, Meng & Zhang, 2019). However, existing prognostic models for OSCC lacks excellent accuracy and a comprehensive assessment. A three-mRNA signature (*PLAU*, *CLDN8* and *CDKN2A*) identified by Zhao et al. (2018b) performs with insufficient accuracy (AUC = 0.609) and this model was not assessed by calibration plot. Another prognostic signature was derived from seven DNA methylation CpG sites (*AJAP1*, *SHANK2*, *FOXA2*, *MT1A*, *ZNF570*, *HOXC4*, and *HOXB4*) as a potential reliable indicator for predicting survival in training cohort (AUC = 0.76), while in validation cohort the accuracy and stability of this model needs further improvements (AUC = 0.66 or 0.67) (Shen et al., 2017b). The current study therefore attempted to develop a better prognosis model with comprehensive evaluation.

The Cancer Genome Atlas (TCGA) is a public database with genome sequencing data on 33 tumor types, analysis of which has contributed a tremendous amount to the understanding of potential molecular mechanisms, diagnostic prediction and the prognosis of cancers (Zhang et al., 2019). In this study we developed and validated a prognostic model with 3 mRNAs based on TCGA OSCC RNA-seq data, which showed good performance for 3- and 5-year overall survival (OS). Furthermore, this 3-mRNA signature was demonstrated as the most important and independent factor for risk of OSCC survival compared with other clinical features (age, gender, race, survival time, survival status, tumor grade, pathological stage and TNM stage).

## Materials and Methods

### Data source and differential expression analysis

Level 3 RNA-Seq data consisting of 328 OSCC tissues and 32 normal controls were downloaded from the TCGA data portal (up to December 8, 2018). Related clinical data (age, gender, race, survival time, survival status, tumor grade, pathological stage and TNM stage) were also obtained. All the data concerned in this study are publicly available from the United States National Cancer Institute (https://portal.gdc.cancer.gov/). Raw sequence data was originally derived from IlluminaHiSeq_RNASeq. We used the edgeR package to identify differentially expressed mRNAs (DEmRNAs) between OSCC and normal tissues. False discovery rate (FDR) was used for multiple testing correction of all p-values. Absolute log_2_fold change(FC) ≥ 2 and the FDR < 0.01 were used as cut-off criteria.

### Construction and evaluation of mRNA prediction model

Only patients with complete information to build prediction model, including age, gender, race, survival time, survival status, pathological stage and TNM stage were included. Metastatic stages were not analyzed, since most data for this item were missing. After filtering available data, a total of 259 OSCC patients and 32 normal tissues were included in our analysis; their baseline information is shown in Table 1.

**Table 1:** Clinicopathological characteristics of 259 patients with oral squamous cell carcinoma.

Characteristic	Subtype	No. of cases (%)
Age (years)	<50	41 (15.83)
	≥50, <60	69 (26.64)
	≥60, <70	88 (33.98)
	≥70	61 (23.55)
Gender	Male	179 (69.11)
	Female	80 (30.89)
Race	White	232 (89.58)
	Asian	9 (3.47%)
	Black or African American	18 (6.95)
Histologic grade	G1	39 (15.06)
	G2	172 (66.41)
	G3	47 (18.15)
	G4	1 (0.39)
Pathological stage	Stage I	16 (6.18)
	Stage II	36 (13.90)
	Stage III	54 (20.85)
	Stage IV	153 (59.07)
Pathological T	T1	23 (8.88)
	T2	76 (29.34)
	T3	60 (23.17)
	T4	100 (38.61)
Pathological N	N0	113 (43.63)
	N1	45 (17.37)
	N2	99 (38.22)
	N3	2 (0.77)
Vital status	Alive	157 (60.62)
	Dead	102 (39.38)

Univariate Cox analysis was used to estimate the association between the expression level of mRNAs and patient’s OS with *p* < 0.05 set as the cut-off of statistical significance. Then, the least absolute shrinkage and selection operator (LASSO) method (Tibshirani, 1997) was used to further screen out prognostic mRNAs. Finally, stepwise multivariate Cox regression analysis was performed to construct a mRNA-derived prognostic model based on the Akaike information criterion (AIC). After calculating the risk score based on the formula of the chosen model, smooth curve fitting was competed to evaluate the relationship between risk score and survival (Li et al., 2019). If a linear relationship between these measures was found, then the cut-off value was set as the median risk score. Otherwise, segmented regression model and likelihood ratio test were employed to find the threshold. The Kaplan Meier survival curve was completed to assess the survival rates of patients with high/low risk score.

A nomogram was generated to predict the individual’s overall survival at 3 years and 5 years. The time-dependent receiver operating characteristic (ROC) curve and calibration plot were used to assess the performance of the nomogram.

### Validation of the mRNA prediction model

Dataset GSE41613 with 97 OSCC patients was selected as the validation cohort from the Gene Expression Omnibus (GEO, https://www.ncbi.nlm.nih.gov/geo/). The time-dependent ROC curve and calibration plots were created to investigate whether the built model could effectively predict survival in OSCC.

### 3-mRNA signature as an independent and important prognostic factor

To verify that the mRNA signature was independent of other clinical characteristics, univariate and multivariate Cox regression analysis were implemented. First, univariate Cox analysis identified the clinical features that were related to OS of OSCC patients. Then multivariate Cox regression was used to explore whether the mRNA signature could be an independent indicator after adjusting other traits.

Then machine learning (extreme gradient boosting (XGBoost)) analysis (Livne et al., 2018) was used to further identify the importance of the mRNA signature on the prognosis of OSCC compared with other clinical features. The parameters were set as following: booster=gbtree, objective=binary:logistic, max_depth=10, min_child_weight=6.23, subsample=0.75, colsample_bytree=0.99. The XGBoost model has been previous demonstrated to provide state-of-the-art results for a variety of medical applications and has received numerous awards in machine learning (Chen & Guestrin, 2016).

### Functional enrichment analysis

DEmRNAs were identified by edgeR package based on the high/low risk score with a cut-off criteria of absolute log_2_FC ≥ 1 and the FDR < 0.05. Gene Ontology (GO) enrichment analysis was performed using the Database for Annotation Visualization and Integrated Discovery (DAVID) and Kyoto Encyclopedia of Genes and Genomes (KEGG) pathways with clusterProfiler package. A *p* value < 0.05 was considered to represent statistical significance. All statistical analyses were performed using R and EmpowerStats software (http://www.empowerstats.com; X&Y Solutions, Inc, Boston, MA, USA).

## Results

### DEmRNAs in OSCC

**Figure 1:**
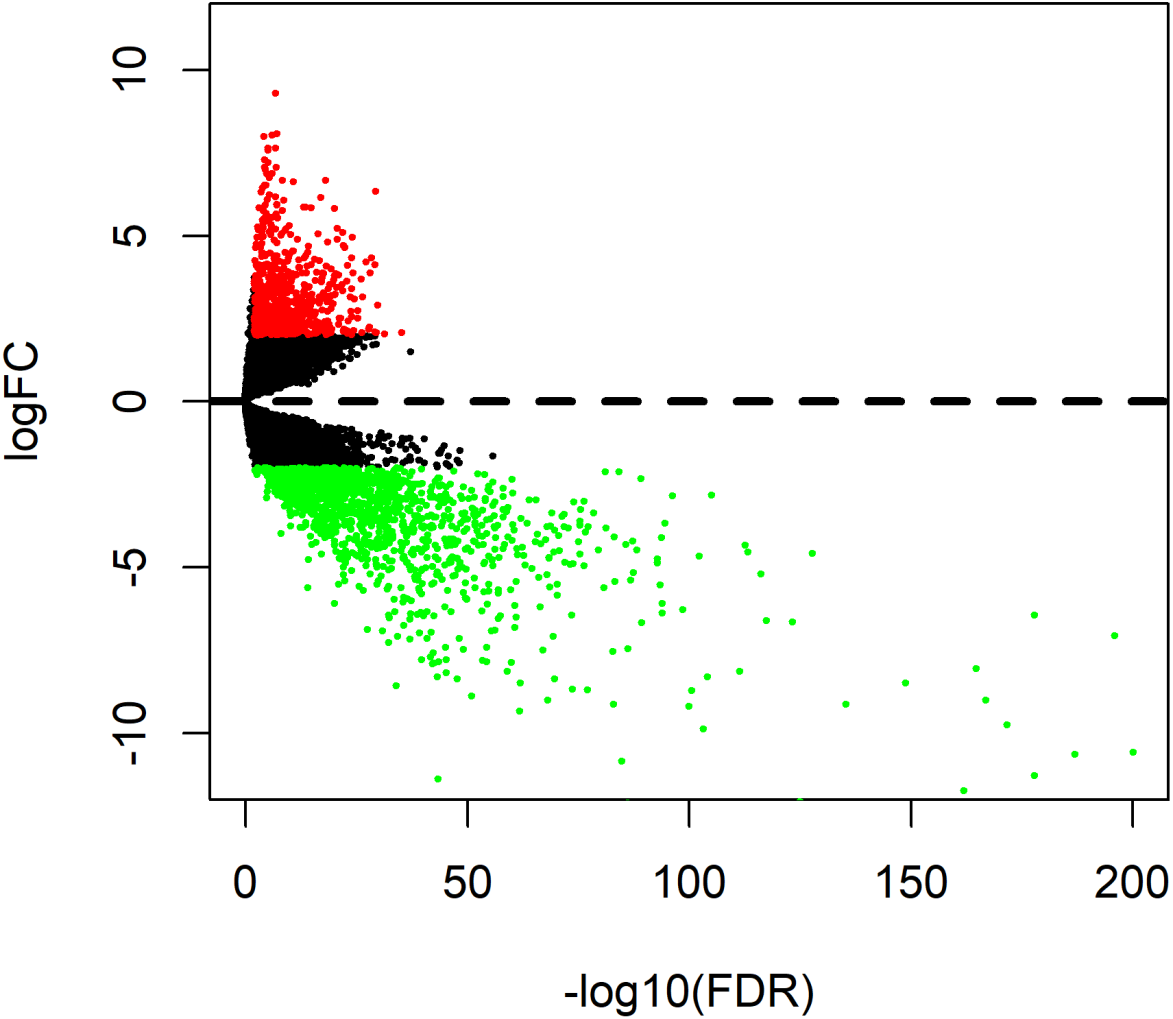
Volcano plot of genesthat are significantly different between OSCC tissues and normal controls. Red spots represent up-regulated genes, and green spots represent down-regulated genes.

A total of 1996 DEmRNAs (707 up-regulated and 1289 down-regulated) were identified in this OSCC dataset (Table S1). The distribution of DEmRNAs between OSCC and normal controls are shown in Fig. 1. The expression heat map of DEmRNAs is presented in Fig. S1 where red and green represents significantly upregulated and downregulated genes, respectively.

### Construction and evaluation of a mRNA prediction model

**Figure 2:**
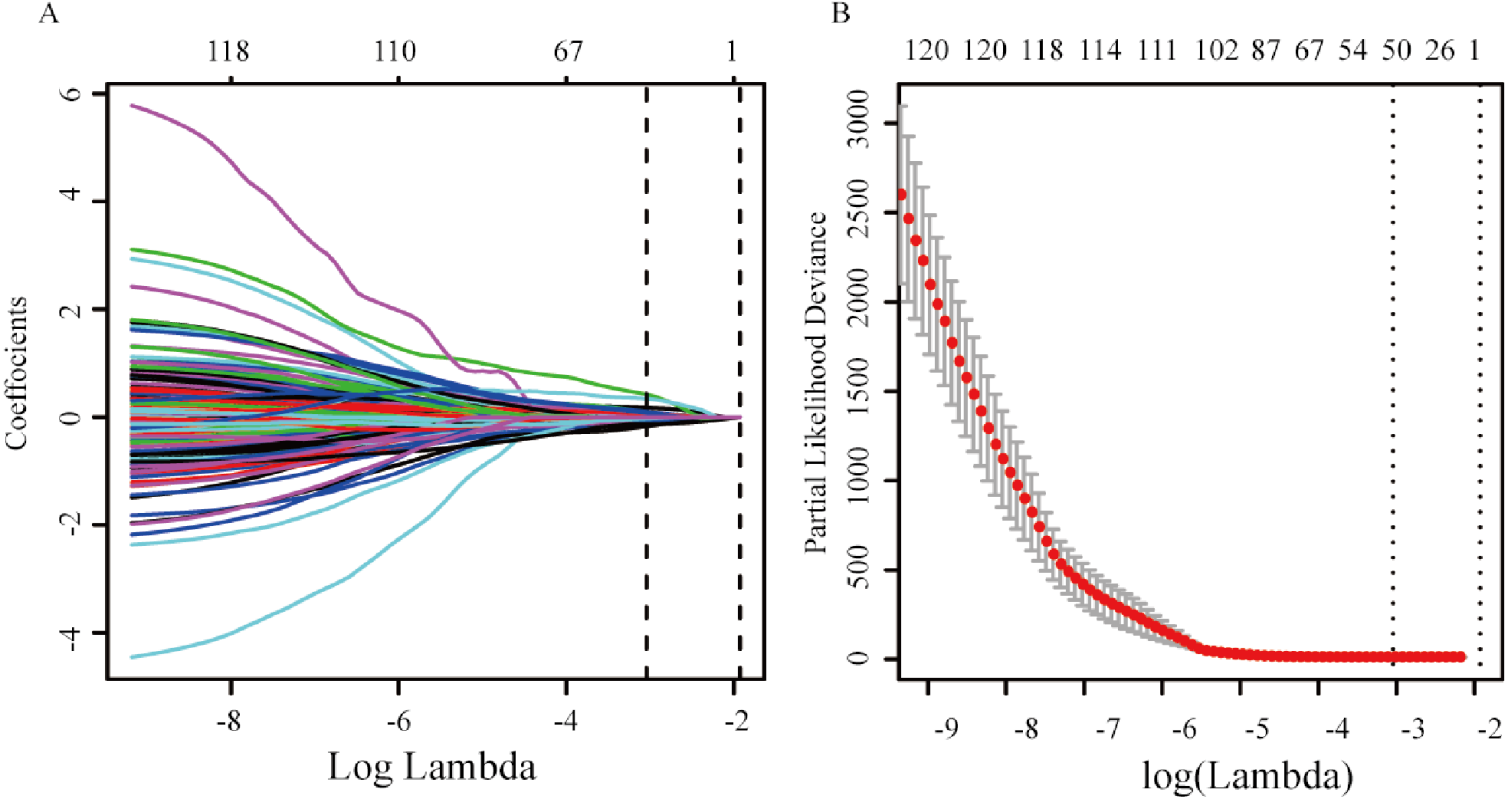
mRNA selection using the least absolute shrinkage and selection operator (LASSO) model. (A) Ten-fold cross-validation for the coefficients of 120 mRNAs in the LASSO model. (B) X-tile analysis of the 50 selected mRNAs.

**Figure 3:**
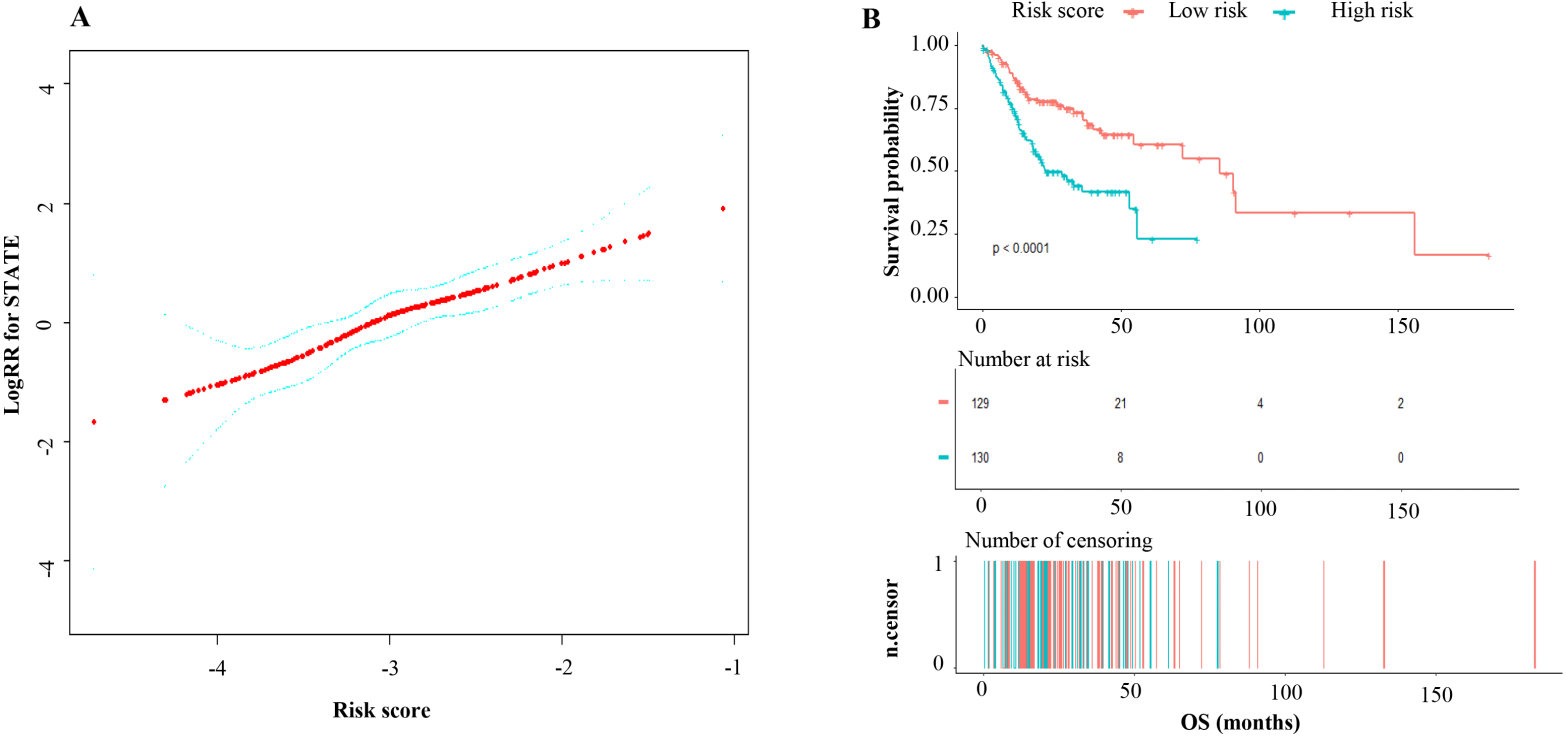
(A) Curve-fitting between the risk score of the 3-mRNA signature and log (risk ratio) for death; (B) Kaplan–Meier analysis of overall survival based on risk score of the 3-mRNAsignature OSCC patients.

A total of 120 DEmRNAs with potential prognostic value were identified by univariate Cox analysis, 50 remained after being filtered by LASSO (Figs. 2A and 2B). Finally, 3 mRNAs (*CLEC3B*, *CLCN1* and *C6*) were selected to construct prediction model by stepwise multivariate Cox regression analysis. The total risk score was imputed as follows: (−0.38602 × expression level of *CLEC3B*) + (−0.20632 × expression level of *CLCN1*) + (0.31541 × expression level of *C6*). The result of smooth curve fitting shows a linear relationship between risk score and survival, thus the median of the risk score was viewed as cut-off value (high risk score: risk score >−3.03, low risk score: risk score <−3.03). Patients with a high risk score had a poor prognosis (Fig. 3).

**Figure 4:**
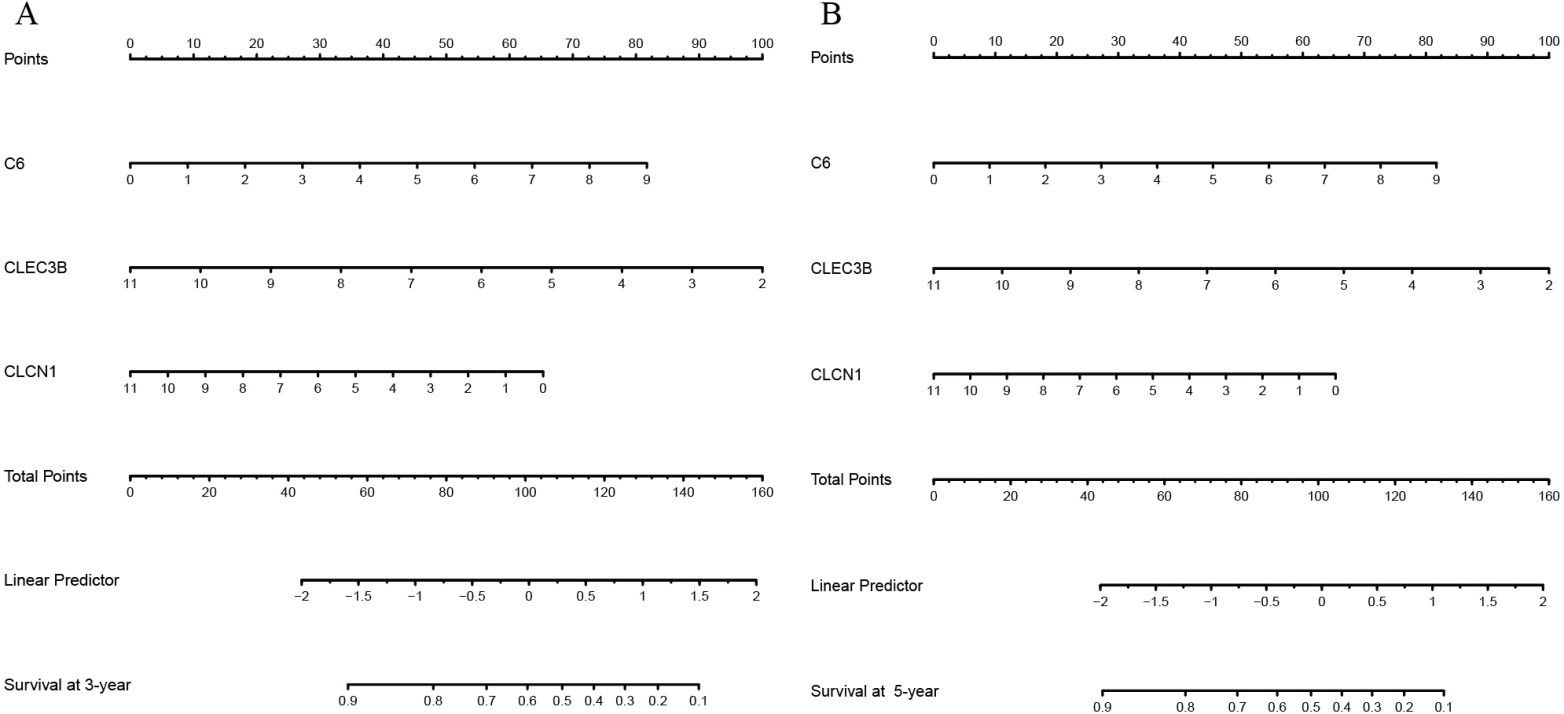
(A) The nomogram for predicting probabilities of patients 3-year overall survival; (B) the nomogram for predicting probabilitiesof patients 5-year overall survival.

**Figure 5:**
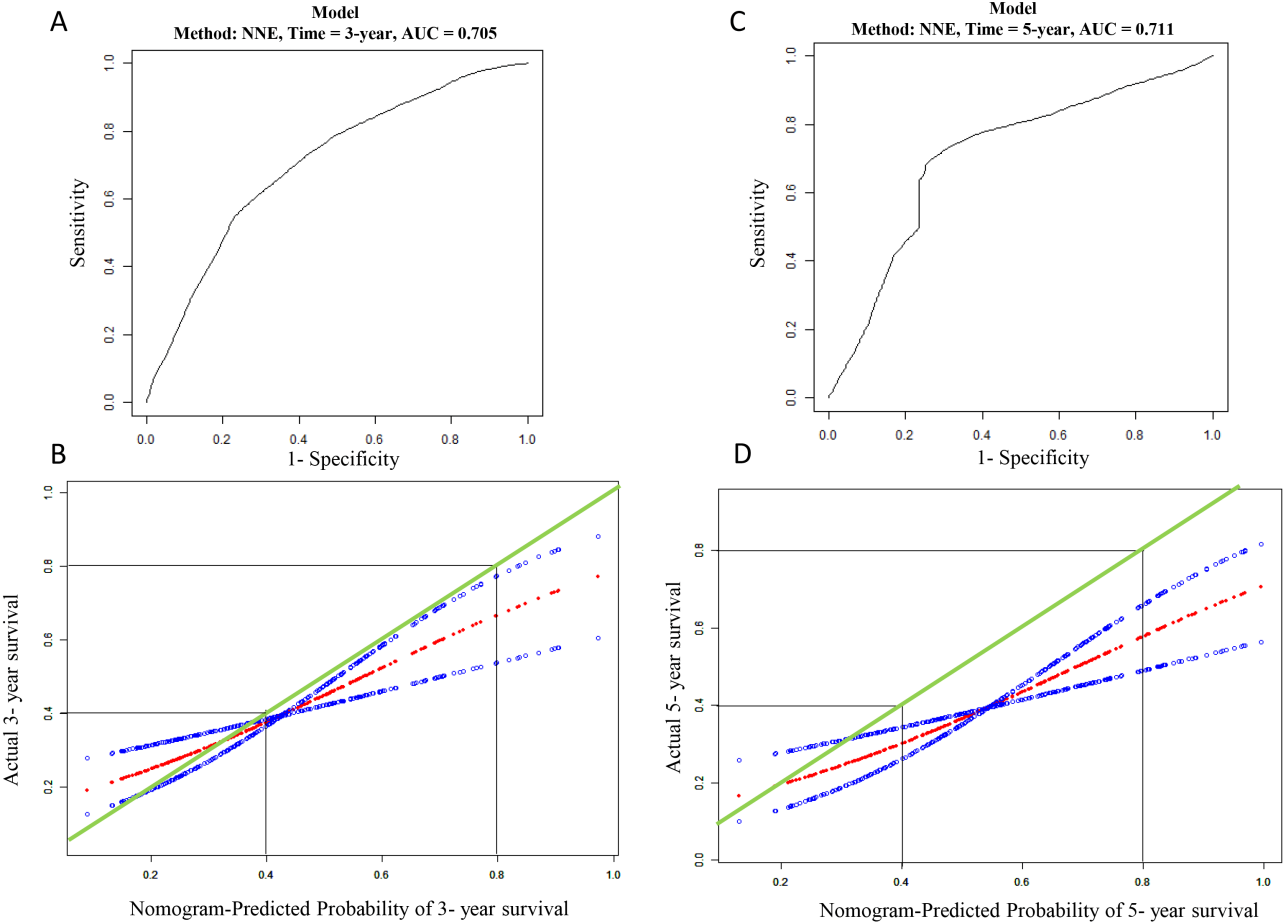
Evaluation of mRNA prediction model. (A) ROC curve based on the mRNA risk score for 3-year overall survival probability in TCGA cohort. (B) The calibration plots for predicting patient 3-year overall survival. Green line indicates actual survival in TCGA cohort; dotted red and blue indicate nomogram-predicted probability of survival and its corresponding 95% confidence interval, respectively. (C) ROC curve based on the mRNA risk score for 5-year in TCGA cohort; (D) the calibration plots for predicting patient 5-year overall survival in TCGA cohort. Green line indicates actual survival in TCGA cohort; dotted red and blue indicate nomogram-predicted probability of survival and its corresponding 95% confidence interval, respectively.

Nomograms of 3- and 5-year OS in the cohort are presented in Fig. 4. AUC of the time-dependent ROC curve was 0.705 and 0.711 for 3- and 5-year survival, respectively (Fig. 5). Remarkable, the calibration curve also demonstrated satisfactory agreement between predictive values and observation values in the probabilities of 3- and 5-year survival (Fig. 5).

### External validation set and performance

**Figure 6:**
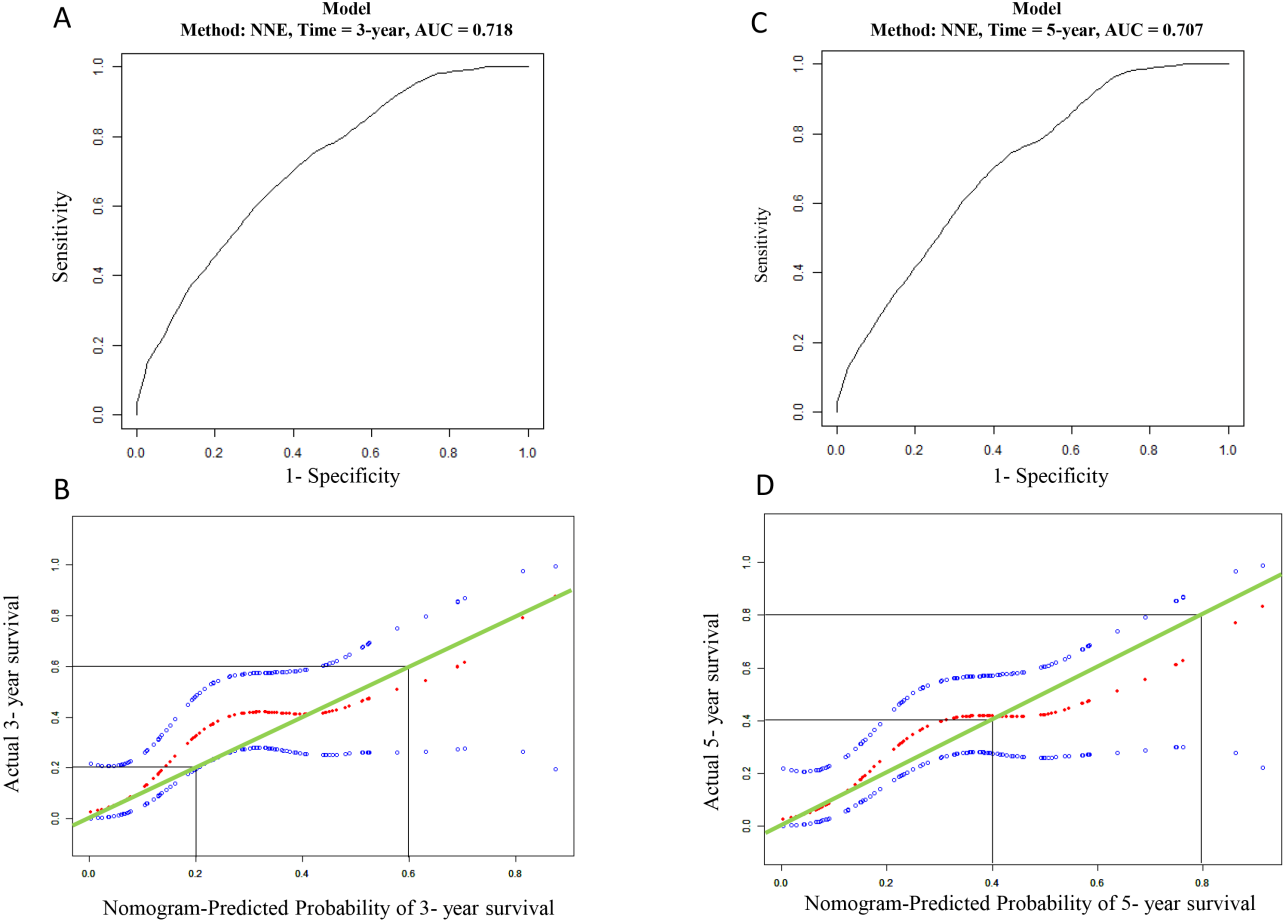
Validation for the mRNA prediction model. (A) ROC curve based on the mRNA risk score for 3-year overall survival probability in validation cohort. (B) The calibration plots for predicting patient 3-year overall survival in validation cohort. Green line indicates actual survival; dotted red and blue indicate nomogram-predicted probability of survival and its corresponding 95% confidence interval, respectively. (C) ROC curve based on the mRNA risk score for 5-year in validation cohort; (D) the calibration plots for predicting patient 5-year overall survival in validation cohort. Green line indicates actual survival; dotted red and blue indicate nomogram-predicted probability of survival and its corresponding 95% confidence interval, respectively.

The GSE41613 dataset including 97 OSCC patient records was chosen to validate the prognostic three genes signature. Time-dependent AUCs of risk scores were 0.718 and 0.717 for 3- and 5-year OS, respectively (Fig. 6). The calibration curve also shows satisfactory agreement between predictive values and observation values for the probabilities of 3- and 5-year OS in the cohort (Fig. 6).

### Risk score of 3-mRNA signature as an independent indicator to predict OSCC prognosis

Cox analysis was performed to assess the independent prognostic value of the 3-mRNA signature and the results are shown in Table 2. According to the results from univariate analysis, the risk score, tumor grade, tumor size, lymphatic metastasis and pathological stage were significantly associated with OS in the TCGA cohort. After multivariate adjustment using the factors above, the risk score remained a powerful and independent prognostic factor (hazard ratio [HR] = 1.9, *p* < 0.001) in this cohort.

**Table 2:** The risk score generated from the 3-mRNA signature as an independent indicator according to Cox proportional hazards regression model.

Factors	Subgroup	Univariate analysis			Multivariate analysis		
		Hazard ratio	95% CI	*p*	Hazard ratio	95% CI	*p*
Age (year)	<50	1					
	≥50, <60	0.8	0.4–1.5	0.516			
	≥60, <70	1	0.5–1.8	0.925			
	>70	1.6	0.9–3.0	0.125			
Gender	Male	1					
	Female	1.1	0.7–1.6	0.722			
Race	White	1					
	Asian	0.7	0.2–2.7	0.561			
	Black or African American	1.9	0.9–3.9	0.086			
Histologic grade	G1	1			1		
	G2	1.7	0.9–3.2	0.121	1.3	0.7–2.6	0.405
	G3	2.2	1.1–4.5	0.035	1.8	0.9–3.8	0.111
	G4	0	0.0–Inf	0.996	0	0.0–Inf	0.996
Pathological stage	Stage I	1			1		
	Stage II	1.5	0.3–7.3	0.604	1	0.1–13.6	0.986
	Stage III	3.4	0.8–14.4	0.1	2.3	0.2–27.8	0.518
	Stage IV	5.1	1.2–20.8	0.024	1.9	0.1–23.8	0.6636
Pathological T	T1	1			1		
	T2	2.1	0.6–7.1	0.23	1.6	0.2–12.5	0.647
	T3	4.8	1.5–15.8	0.009	2.3	0.3–17.7	0.411
	T4	4.4	1.4–14.2	0.013	2.2	0.3–17.2	0.45
Pathological N	N0	1			1		
	N1	0.9	0.5–1.8	0.813	0.6	0.3–1.3	0.217
	N2	2.2	1.4–3.3	<0.001	1.3	0.7–2.4	0.447
	N3	4.2	1.0–17.5	0.05	3.5	0.8–15.4	0.099
Risk scores	Low risk	1			1		
	High risk	2.3	1.5–3.6	<0.001	1.9	1.2–2.9	0.004

**Figure 7:**
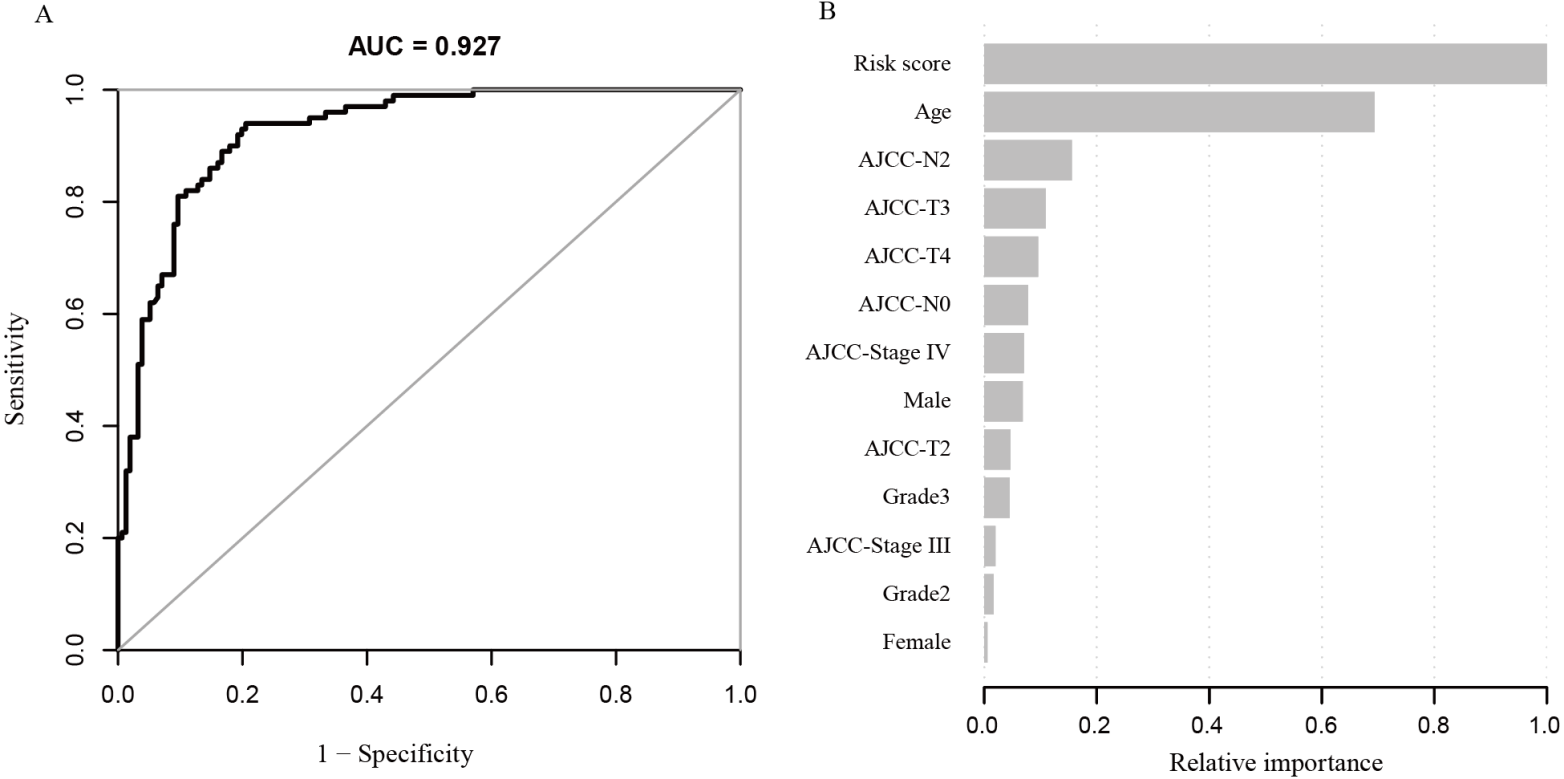
(A) ROC curve based on the modelconstructed by machine learning; (B) risk factors contribution to deathprediction.

To further understand the importance of the risk score, machine learning analysis was carried out. The XGBoost model indicated a high performance in predicting OSCC patients’ survival status (AUC = 0.927) (Fig. 7). In addition, the most important parameter for final survival status prediction was identified with this model as the risk score (Table 3 and Fig. 7).

**Table 3:** Parameters contribution to final survival status prediction.

Feature	Gain	Relative importance
Risk scores	0.4158	100%
Age	0.2885	69.38%
Pathological N: 2	0.0647	15.56%
Pathological T: 3	0.0454	10.92%
Pathological T: 4	0.0399	9.60%
Pathological N: 0	0.0325	7.82%
Pathological stage: IV	0.0292	7.02%
Gender: Male	0.0285	6.85%
Pathological T: 2	0.0194	4.67%
Histologic grade: G3	0.0187	4.50%
Pathological stage: 3	0.0083	2.00%
Histologic grade: G2	0.0069	1.66%
Gender: Female	0.0023	0.55%

### Functional enrichment analysis

**Figure 8:**
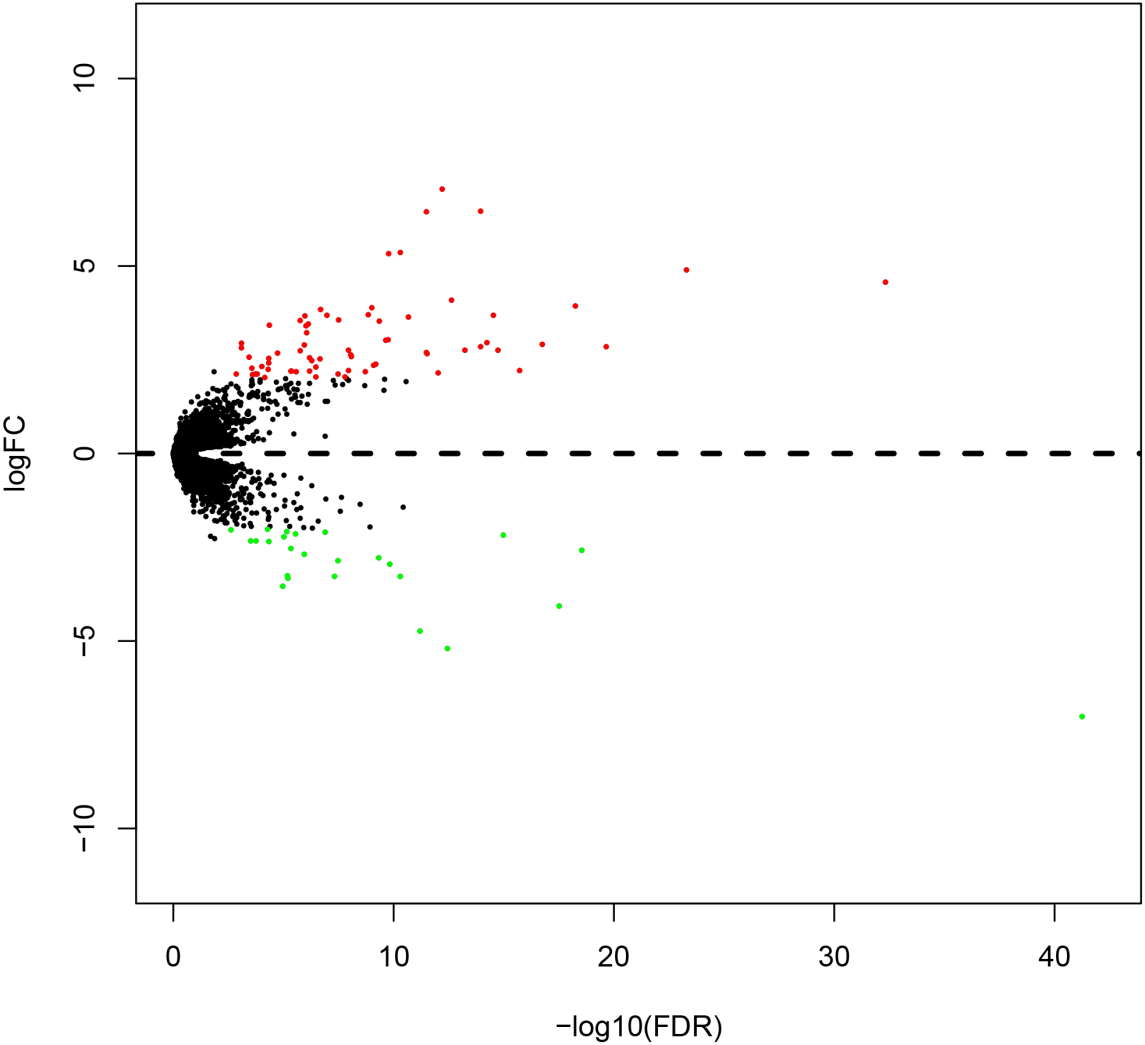
Volcano plot of genes that are significantlydifferent based on the risk score of OSCC patients. Red spots represent up-regulated genes, and green spots represent down-regulated genes.

**Figure 9:**
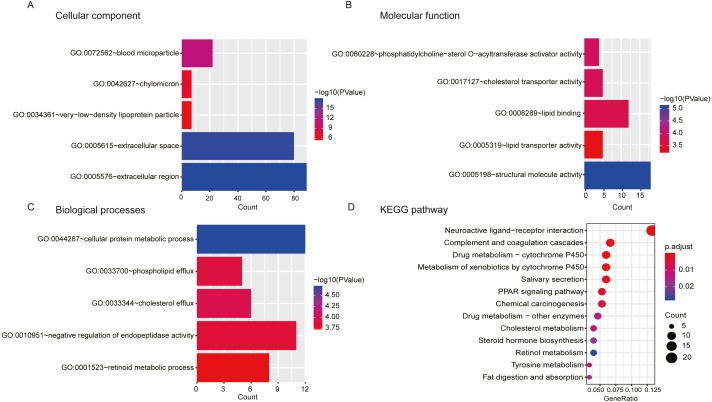
GO and KEGG pathway analyses. (A, B, C) GO analyses of the differentially expressed mRNAs based on the risk score of OSCC. (D) KEGG analysis of the differentially expressed mRNAs based on the risk of OSCC.

A total of 404 differentially expressed genes (218 upregulated genes and 186 downregulated genes) correlated with high/low risk score group were displayed (Fig. 8). To better understand the potential mechanism of processing different risk score groups, GO and KEGG enrichment analyses were performed. For “biological processes (BP)”, the top five enriched terms were cellular protein metabolic process, negative regulation of endopeptidase activity, phospholipid efflux, retinoid metabolic process and cholesterol efflux. For the “cellular component (CC)” ontology, the top five were: extracellular space, extracellular region, blood microparticle, chylomicron and very-low-density lipoprotein particle. Finally, the five “molecular function (MF)” top terms were structural molecule activity, lipid binding, cholesterol transporter activity, phosphatidylcholine-sterol O-acyltransferase activator activity and lipid transporter activity (Figs. 9A, 9B and 9C and Table S2).

Additionally, a total of 13 significantly enriched KEGG pathways are listed in Table S3, and the top 10 KEGG pathways are shown in Fig. 9D. A neuroactive ligand–receptor interaction was found to be the main associated pathway.

## Discussion

In the era of precision medicine, it is necessary to use accurate prognostic models to guide the clinical decision-making process and to design a more personalized therapeutic program for patients. However, the performance of existing OSCC prognostic models are limited. Thus, this study aimed to develop a better prognostic model. A 3-mRNA-based model (*CLEC3B*, *CLCN1* and *C6*) of OSCC and it showed moderate predictive ability (training cohort: AUC = 0.705 and 0.711 for 3- and 5-year OS; validation cohort: AUC = 0.718 and 0.717 for 3- and 5-year OS). In addition, the 3-mRNA signature was an independent and the most important risk factor of the prognosis of OSCC.

Three articles in the literature build a prognostic model of OSCC based on molecule. A 3-mRNA signature and a 3-lncRNA signature indicate an imperfect predictive ability (AUC < 0.7) (Zhao et al., 2018a; Zhao et al., 2018b). Although seven DNA methylation CpG sites are a potential reliable indicator for predicting OS (AUC = 0.76), its accuracy for validation requires improvement (AUC = 0.66 or 0.67) (Shen et al., 2017b). The 3-mRNA-based model constructed in the current study performed well in both training and validation cohorts (training cohort: 0.711; validation cohort: AUC = 0.717). Calibration is also an important indicator to assess prediction model; however, the three exiting models fail to perform calibration curve (Shen et al., 2017b; Zhao et al., 2018a; Zhao et al., 2018b). To assess the consistency of our prediction model, calibration curves were implemented and the result of analysis indicated that our model performed well.

In addition, to test the importance of survival status prediction with this 3-mRNA signature, machine learning method (XGBoost) was used to rank clinical parameters and the 3-mRNA signature. XGBoost is a gradient tree boosting algorithm with best performance to solve the classification problems. (Ogunleye & Qing-Guo, 2019). In addition, XGBoost has the advantage of assessing of parameter importance by calculating as the cumulative average of the modality gain over all the constituent decision trees in the ensemble model (Livne et al., 2018). The results of machine learning verified that 3-mRNA signature was the most important factor to OSCC survival with high accuracy (AUC = 0.927). This finding again demonstrated that the identified 3-mRNA signature could better predict the survival for OSCC patients and they may play important roles in the development of OSCC.

*CLEC3B*, a member of the C-type lectin superfamily, is located on human chromosome 3p21.31. The function of *CLEC3B* depends on the location of the tumor. The gene acts as an oncogene in colorectal cancer because *CLEC3B* is secreted by cancer-associated fibroblasts promotes tumor cell migration (Zhu et al., 2019), whereas it could inhibit clear cell renal cell carcinoma proliferation via mitogen-activated protein kinase (MAPK) pathway (Liu et al., 2018). In our study, it may act as a tumor suppressor in that down-regulation of *CLEC3B* indicates a poor prognosis. *CLEC3B* expression has negative correlation with proliferation inducers and proliferative markers, while there is a positive correlation between *CLEC3B* and proliferation inhibitors, indicating *CLEC3B* lowers cell proliferation, and may explain why it may serve as a tumor suppressor in OSCC (Liu et al., 2018). In addition, tetranectin coded by CLEC3B is significantly down-regulation in metastatic OSCC (Arellano-Garcia et al., 2010), indicating that CLEC3B may suppress OSCC development. However, specific mechanism of CLEC3B needs to be further explored.

Similarly, *C6*, complement 6, encodes protein which formats the membrane attack complex (MAC) with C5b, C7, C8 and C9. *C6* is down-regulated in oesophageal carcinoma and its reduction may result in a reduction in local complement components and MAC and thus contribute to cancer resistance to the complement attack (Oka et al., 2001). When tumor cells acquire resistance to complement attack, the deposition of sublytic MAC on the cell membrane is increased. Sublytic doses of the MAC involved in various biological processes of tumor, such as the promotion of angiogenesis, proliferation, differentiation and the inhibition of apoptosis (Pio, Corrales & Lambris, 2014). That is a partial explanation why *C6* was down-regulated in OSCC but its high expression indicated a poor prognosis.

*CLCN1* is a member of chloride channel genes family, encoding voltage-gated chloride channel (CLC-1). Previous studies have indicated that CLC-1 determines up to 80% of the resting membrane conductance in skeletal muscles and the mutation of *CLCN1* reduces chloride conductance and then result in myotonia congenita (Peng et al., 2018; Tsujino et al., 2011). However, the function of *CLCN1* in tumors has not been studied. Its down-regulation was observed to indicate a poor prognosis. This shows that *CLCN1* may serve as a tumor suppressor gene in OSCC which may be because chloride channels are involved in the regulation of cell cycle (Peretti et al., 2015).

GO enrichment analysis indicated that the 3-mRNA signature may be involved in lipid metabolism. Increasing studies have found that lipid metabolism plays an important role in tumor development, including tumor cell migration (Byon et al., 2009), proliferation and angiogenesis (Lu et al., 2017). In addition, lipid metabolism is associated with oxidative stress which also influences tumor progression (Zablocka-Slowinska et al., 2019). Neuroactive ligand–receptor interaction was found to be the most significant pathway in this study, which participates in multiple biological processes, such as apoptosis and cell proliferation (Zan & Li, 2019).

This study identified three valuable mRNAs that are associated with the prognosis of OSCC. The 3-mRNA signature showed satisfactory performance in both training and validation cohort, however, it is still needed to be further validated in cohort study. In addition, biological function of the three prognostic value genes needs to be explored.

## Conclusion

A novel 3-mRNA signature (*CLEC3B*, *C6* and *CLCN1*) successful predicted the survival of OSCC patients in both training and test cohort. In addition, the signature is the independent and the most important risk factor of OSCC.

##  Supplemental Information

10.7717/peerj.7360/supp-1 Table S1 The list of DEmRNAs

10.7717/peerj.7360/supp-2 Table S2 Geneontology analyses of the differentially expressed mRNAs based on the risk score

10.7717/peerj.7360/supp-3 Table S3 KEGG analyses of the differentially expressed mRNAs based on the risk score

10.7717/peerj.7360/supp-4 Table S4 The baseline of validation cohort

10.7717/peerj.7360/supp-5 Figure S1 Heatmap of DEmRNAs

10.7717/peerj.7360/supp-6 Supplemental Information 6 RNA-seq from TCGA

10.7717/peerj.7360/supp-7 Supplemental Information 7 Validation cohort data

## Additional Information and Declarations

### Data Availability

The following information was supplied regarding data availability: The raw measurements are available in the Supplemental Files.

## References

[ref-1] Arellano-Garcia ME, Li R, Liu X, Xie Y, Yan X, Loo JA, Hu S (2010). Identification of tetranectin as a potential biomarker for metastatic oral cancer. International Journal of Molecular Sciences.

[ref-2] Boxberg M, Bollwein C, Johrens K, Kuhn PH, Haller B, Steiger K, Wolff KD, Kolk A, Jesinghaus M, Weichert W (2018). Novel prognostic histopathological grading system in oral squamous cell carcinoma based on tumour budding and cell nest size shows high interobserver and intraobserver concordance. Journal of Clinical Pathology.

[ref-3] Byon CH, Hardy RW, Ren C, Ponnazhagan S, Welch DR, McDonald JM, Chen Y (2009). Free fatty acids enhance breast cancer cell migration through plasminogen activator inhibitor-1 and SMAD4. Laboratory Investigation.

[ref-4] Chen T, Guestrin C (2016). XGBoost: a scalable tree boosting system.

[ref-5] Feng G, Ma HM, Huang HB, Li YW, Zhang P, Huang JJ, Cheng L, Li GR (2019). Overexpression of COL5A1 promotes tumor progression and metastasis and correlates with poor survival of patients with clear cell renal cell carcinoma. Cancer Management and Research.

[ref-6] Lee SY, Hwang I, Park YS, Gardner J, Ro JY (2010). Metastatic lymph node ratio in advanced gastric carcinoma: a better prognostic factor than number of metastatic lymph nodes?. International Journal of Oncology.

[ref-7] Li W, Shi D, Song W, Xu L, Zhang L, Feng X, Lu R, Wang X, Meng H (2019). A novel U-shaped relationship between BMI and risk of generalized aggressive periodontitis in Chinese: a cross-sectional study. Journal of Periodontology.

[ref-8] Liu J, Liu Z, Liu Q, Li L, Fan X, Wen T, An G (2018). CLEC3B is downregulated and inhibits proliferation in clear cell renal cell carcinoma. Oncology Reports.

[ref-9] Livne M, Boldsen JK, Mikkelsen IK, Fiebach JB, Sobesky J, Mouridsen K (2018). Boosted tree model reforms multimodal magnetic resonance imaging infarct prediction in acute stroke. Stroke.

[ref-10] Lu CW, Lo YH, Chen CH, Lin CY, Tsai CH, Chen PJ, Yang YF, Wang CH, Tan CH, Hou MF, Yuan SF (2017). VLDL and LDL, but not HDL, promote breast cancer cell proliferation, metastasis and angiogenesis. Cancer Letters.

[ref-11] Ogunleye AA, Qing-Guo W (2019). XGBoost model for chronic kidney disease diagnosis. IEEE/ACM Transactions on Computational Biology and Bioinformatics.

[ref-12] Oka R, Sasagawa T, Ninomiya I, Miwa K, Tanii H, Saijoh K (2001). Reduction in the local expression of complement component 6 (C6) and 7 (C7) mRNAs in oesophageal carcinoma. European Journal of Cancer.

[ref-13] Peng YJ, Lee YC, Fu SJ, Chien YC, Liao YF, Chen TY, Jeng CJ, Tang CY (2018). FKBP8 enhances protein stability of the CLC-1 chloride channel at the plasma membrane. International Journal of Molecular Sciences.

[ref-14] Peretti M, Angelini M, Savalli N, Florio T, Yuspa SH, Mazzanti M (2015). Chloride channels in cancer: focus on chloride intracellular channel 1 and 4 (CLIC1 AND CLIC4) proteins in tumor development and as novel therapeutic targets. Biochimica et Biophysica Acta/General Subjects.

[ref-15] Pio R, Corrales L, Lambris JD (2014). The role of complement in tumor growth. Advances in Experimental Medicine and Biology.

[ref-16] Shao T, Huang J, Zheng Z, Wu Q, Liu T, Lv X (2018). SCCA, TSGF, and the long non-coding RNA AC007271.3 are effective biomarkers for diagnosing oral squamous cell carcinoma. Cellular Physiology and Biochemistry.

[ref-17] Shen S, Bai J, Wei Y, Wang G, Li Q, Zhang R, Duan W, Yang S, Du M, Zhao Y, Christiani DC, Chen F (2017a). A seven-gene prognostic signature for rapid determination of head and neck squamous cell carcinoma survival. Oncology Reports.

[ref-18] Shen S, Wang G, Shi Q, Zhang R, Zhao Y, Wei Y, Chen F, Christiani DC (2017b). Seven-CpG-based prognostic signature coupled with gene expression predicts survival of oral squamous cell carcinoma. Clinical Epigenetics.

[ref-19] Shimomura H, Sasahira T, Nakashima C, Shimomura-Kurihara M, Kirita T (2018). Downregulation of DHRS9 is associated with poor prognosis in oral squamous cell carcinoma. Pathology.

[ref-20] Siegel RL, Miller KD, Jemal A (2017). Cancer Statistics, 2017. CA: A Cancer Journal for Clinicians.

[ref-21] Tian S, Meng G, Zhang W (2019). A six-mRNA prognostic model to predict survival in head and neck squamous cell carcinoma. Cancer Management and Research.

[ref-22] Tibshirani R (1997). The lasso method for variable selection in the Cox model. Statistics in Medicine.

[ref-23] Tschirdewahn S, Panic A, Pullen L, Harke NN, Hadaschik BH, Riesz P, Horvath A, Szalontai J, Nyirady P, Baba HA, Reis H, Szarvas T (2019). Circulating and tissue IMP3 levels are correlated with poor survival in renal cell carcinoma. International Journal of Cancer.

[ref-24] Tsujino A, Kaibara M, Hayashi H, Eguchi H, Nakayama S, Sato K, Fukuda T, Tateishi Y, Shirabe S, Taniyama K, Kawakami A (2011). A CLCN1 mutation in dominant myotonia congenita impairs the increment of chloride conductance during repetitive depolarization. Neuroscience Letters.

[ref-25] Wang H, Liang X, Li M, Tao X, Tai S, Fan Z, Wang Z, Cheng B, Xia J (2017). Chemokine (CC motif) ligand 18 upregulates Slug expression to promote stem-cell like features by activating the mammalian target of rapamycin pathway in oral squamous cell carcinoma. Cancer Science.

[ref-26] Zablocka-Slowinska K, Placzkowska S, Skorska K, Prescha A, Pawelczyk K, Porebska I, Kosacka M, Grajeta H (2019). Oxidative stress in lung cancer patients is associated with altered serum markers of lipid metabolism. PLOS ONE.

[ref-27] Zan XY, Li L (2019). Construction of lncRNA-mediated ceRNA network to reveal clinically relevant lncRNA biomarkers in glioblastomas. Oncology Letters.

[ref-28] Zhang S, Cao R, Li Q, Yao M, Chen Y, Zhou H (2019). Comprehensive analysis of lncRNA-associated competing endogenous RNA network in tongue squamous cell carcinoma. PeerJ.

[ref-29] Zhao C, Zou H, Wang J, Shen J, Liu H (2018a). A three long noncoding RNA-based signature for oral squamous cell carcinoma prognosis prediction. DNA and Cell Biology.

[ref-30] Zhao X, Sun S, Zeng X, Cui L (2018b). Expression profiles analysis identifies a novel three-mRNA signature to predict overall survival in oral squamous cell carcinoma. American Journal of Cancer Research.

[ref-31] Zhu HF, Zhang XH, Gu CS, Zhong Y, Long T, Ma YD, Hu ZY, Li ZG, Wang XY (2019). Cancer-associated fibroblasts promote colorectal cancer progression by secreting CLEC3B. Cancer Biology & Therapy.

