# Supplementary material for: A 3-mRNA-based prognostic signature of survival in oral squamous cell carcinoma
Source: PeerJ. 2019 Jul 31;7:e7360. doi: 10.7717/peerj.7360 (PMC6679650; doi:10.7717/peerj.7360)
Supplement: Table S2 [file peerj-07-7360-s002.docx]

**Supplementary Table S2**

Gene ontology analyses of the differentially expressed mRNAs between high risk score and low risk score.

| Category | Term | Count | % | PValue | Genes | Benjamini |
| --- | --- | --- | --- | --- | --- | --- |
| GOTERM_MF_DIRECT | GO:0005198~structural molecule activity | 18 | 4.5 | 8.00E-06 | LOR, GFAP, KRT13, KRT32, KRT9, KRT81, FGG, KRT27, FGA, FGB, FLG, KRT7, KRTAP3-2, KRT78, SPRR3, KRT3, KRT4, KRT83 | 0.003544 |
| GOTERM_MF_DIRECT | GO:0008289~lipid binding | 12 | 3 | 1.95E-04 | APOA4, APOA2, BPIFB2, APOA1, PLIN1, APOC3, BPIFB6, APOA5, APOH, ACOT12, FABP1, MAL | 0.021446 |
| GOTERM_MF_DIRECT | GO:0017127~cholesterol transporter activity | 5 | 1.25 | 1.67E-04 | APOA4, APOA2, APOB, APOA1, APOA5 | 0.024415 |
| GOTERM_MF_DIRECT | GO:0060228~phosphatidylcholine-sterol O-acyltransferase activator activity | 4 | 1 | 1.43E-04 | APOA4, APOA2, APOA1, APOA5 | 0.03125 |
| GOTERM_MF_DIRECT | GO:0005319~lipid transporter activity | 5 | 1.25 | 5.49E-04 | APOA4, APOA2, APOB, SFTPA1, ABCA3 | 0.047557 |
| GOTERM_CC_DIRECT | GO:0005615~extracellular space | 80 | 20 | 2.22E-18 | PRH2, C6ORF58, CRP, HP, VTN, SPINK8, SHH, KRT81, APOA4, TTR, IFNL1, APOB, APOA1, CST11, IFNL2, APOA5, TDGF1, APOH, LBP, IFNK, KRT83, MUC13, IL13RA2, KNG1, CRISP3, CTRB1, AMBP, SCGB2A1, UCN3, CBLN2, SCGB1D2, SCGB3A1, SMR3B, GC, MIA, BPIFB2, SPOCK3, PRTN3, RBP3, ENDOU, CXCL8, SFTPA1, IL33, ABCA3, TAC3, KRT9, CALCA, ZG16B, ARG1, CCL25, FGG, FGA, ALB, FGB, APOC3, TFF3, KRT2, SFTPC, SERPINB12, TFF1, SFTPB, LPO, KLK3, FETUB, C4BPA, OPRPN, ADIPOQ, PLG, SERPINI2, ORM1, LCN1, BMPER, NPY, SFTPA2, KRT78, IGFL1, CP, SST, CSN3, MUC5B | 2.57E-16 |
| GOTERM_CC_DIRECT | GO:0005576~extracellular region | 89 | 22.25 | 1.44E-18 | CGB3, CRP, OBP2B, HP, VTN, HTN3, SHH, SPINK7, SPINK9, APOA4, TTR, IFNL1, HTN1, APOB, APOA2, APOA1, IFNL2, ITIH1, RSPO2, APOA5, APOH, IFNK, LBP, HHIP, FGF3, IL13RA2, FGF4, KNG1, CRISP3, PRG4, CTRB1, ZP4, COLEC10, PKDCC, PSG1, COLEC11, C8G, AMBP, C4ORF26, PSG5, C1QL2, SCGB3A2, SMR3A, GC, C7, ENPP6, TSPEAR, DEFB124, C6, RBP3, BPIFB6, ENDOU, CXCL8, SFTPA1, IL33, TAC3, CALCA, CCL25, COL9A1, FGG, FGA, BCHE, FGB, ALB, GALP, APOC3, TFF3, SFTPC, SFTPB, ANGPTL5, PRB2, KLK3, FETUB, C4BPA, OPRPN, MUC6, ADIPOQ, HPR, PLG, ORM1, LCN1, BMPER, COL19A1, NPY, PENK, SFTPA2, CP, SST, CSN3 | 3.35E-16 |
| GOTERM_CC_DIRECT | GO:0072562~blood microparticle | 22 | 5.5 | 3.96E-12 | GC, KNG1, HP, VTN, C4BPA, PLG, HPR, C8G, APOA4, AMBP, ORM1, FGG, APOA2, APOA1, FGA, ALB, FGB, ITIH1, BCHE, APOA5, IGLL1, CP | 3.06E-10 |
| GOTERM_CC_DIRECT | GO:0042627~chylomicron | 7 | 1.75 | 1.71E-07 | APOA4, APOA2, APOB, APOA1, APOC3, APOA5, APOH | 9.89E-06 |
| GOTERM_CC_DIRECT | GO:0034361~very-low-density lipoprotein particle | 7 | 1.75 | 1.99E-06 | APOA4, APOA2, APOB, APOA1, APOC3, APOA5, APOH | 9.21E-05 |
| GOTERM_CC_DIRECT | GO:0070062~extracellular exosome | 89 | 22.25 | 1.15E-05 | ALDH8A1, ARSF, C6ORF58, CRP, AQP5, HP, VTN, SLC22A11, APOA4, TTR, APOB, APOA2, APOA1, ITIH1, APOH, IGLL1, LBP, TREH, PI16, MUC13, KRT84, KNG1, CRISP3, CLCA4, KRT13, ACTN3, LRRC26, C8G, AMBP, DHRS2, RHCG, SPRR3, CTSE, PSCA, SCGB3A1, SMR3B, GC, FXYD2, C7, ENPP6, BPIFB2, PRTN3, C6, ITLN1, ALDOB, CRNN, PSMA8, TAC3, KRT9, ZG16B, ARG1, FGG, KRT27, FGA, GPM6A, CD177, FGB, ALB, CRB2, KRT7, UPK1A, APOC3, TFF3, KRT2, TGM3, SERPINB12, KRT3, GSTA2, LPO, GSTA3, MUC21, PRR27, KLK3, UPB1, FETUB, ADIPOQ, HPR, PLG, KRT32, SERPINI2, ORM1, LCN1, KRT36, NPHS2, KRT78, FABP1, CP, MUC5B, UGT2B7 | 4.44E-04 |
| GOTERM_CC_DIRECT | GO:0005882~intermediate filament | 12 | 3 | 1.81E-05 | KRT9, GFAP, KRT36, KRT27, FLG, KRT7, KRT13, KRT2, KRT3, KRT4, KRT20, KRT32 | 6.00E-04 |
| GOTERM_BP_DIRECT | GO:0044267~cellular protein metabolic process | 12 | 3 | 1.99E-05 | CALCA, APOA4, TTR, APOA1, FGA, KLK3, SFTPA2, SFTPC, SFTPA1, ABCA3, PLG, SFTPB | 0.030512 |
| GOTERM_BP_DIRECT | GO:0010951~negative regulation of endopeptidase activity | 11 | 2.75 | 1.31E-04 | AMBP, KNG1, LCN1, SPOCK3, ITIH1, CRB2, FETUB, SERPINB12, VTN, OPRPN, SERPINI2 | 0.033496 |
| GOTERM_BP_DIRECT | GO:0033700~phospholipid efflux | 5 | 1.25 | 1.23E-04 | APOA4, APOA2, APOA1, APOC3, APOA5 | 0.037445 |
| GOTERM_BP_DIRECT | GO:0001523~retinoid metabolic process | 8 | 2 | 1.80E-04 | APOA4, TTR, APOA2, APOB, APOA1, RBP3, APOC3, RLBP1 | 0.039174 |
| GOTERM_BP_DIRECT | GO:0033344~cholesterol efflux | 6 | 1.5 | 1.06E-04 | APOA4, APOA2, APOB, APOA1, APOC3, APOA5 | 0.040516 |
| GOTERM_BP_DIRECT | GO:0045109~intermediate filament organization | 5 | 1.25 | 2.16E-04 | KRT9, GFAP, KRT2, KRT20, SHH | 0.041178 |
| GOTERM_BP_DIRECT | GO:0042157~lipoprotein metabolic process | 7 | 1.75 | 8.64E-05 | APOA4, APOA2, APOB, APOA1, ALB, APOC3, APOA5 | 0.043878 |
