# Supplementary material for: A 3-mRNA-based prognostic signature of survival in oral squamous cell carcinoma
Source: PeerJ. 2019 Jul 31;7:e7360. doi: 10.7717/peerj.7360 (PMC6679650; doi:10.7717/peerj.7360)
Supplement: Table S3 [file peerj-07-7360-s003.docx]

**Supplementary Table S3**

KEGG analyses of the differentially expressed mRNAs between high risk score and low risk score.

| ID | Description | GeneRatio | pvalue | p.adjust | qvalue |
| --- | --- | --- | --- | --- | --- |
| hsa04610 | Complement and coagulation cascades | 10/144 | 2.40E-06 | 0.000482 | 0.000449 |
| hsa00982 | Drug metabolism - cytochrome P450 | 9/144 | 8.67E-06 | 0.000872 | 0.000813 |
| hsa00980 | Metabolism of xenobiotics by cytochrome P450 | 9/144 | 1.36E-05 | 0.000911 | 0.000849 |
| hsa04970 | Salivary secretion | 9/144 | 5.36E-05 | 0.002695 | 0.002512 |
| hsa03320 | PPAR signaling pathway | 8/144 | 8.08E-05 | 0.003249 | 0.003029 |
| hsa05204 | Chemical carcinogenesis | 8/144 | 0.000168 | 0.005624 | 0.005243 |
| hsa04979 | Cholesterol metabolism | 6/144 | 0.000365 | 0.010492 | 0.00978 |
| hsa00350 | Tyrosine metabolism | 5/144 | 0.000578 | 0.014532 | 0.013547 |
| hsa00983 | Drug metabolism - other enzymes | 7/144 | 0.000778 | 0.017376 | 0.016198 |
| hsa00140 | Steroid hormone biosynthesis | 6/144 | 0.000983 | 0.019447 | 0.018128 |
| hsa04975 | Fat digestion and absorption | 5/144 | 0.001064 | 0.019447 | 0.018128 |
| hsa00830 | Retinol metabolism | 6/144 | 0.001755 | 0.029399 | 0.027406 |
| hsa04080 | Neuroactive ligand-receptor interaction | 13/144 | 0.002577 | 0.03984 | 0.037138 |
